# Amino acid substitutions in norovirus VP1 dictate host dissemination via variations in cellular attachment

**DOI:** 10.1128/jvi.01719-23

**Published:** 2023-11-30

**Authors:** Jake T. Mills, Susanna C. Minogue, Joseph S. Snowden, Wynter K. C. Arden, David J. Rowlands, Nicola J. Stonehouse, Christiane E. Wobus, Morgan R. Herod

**Affiliations:** 1 Astbury Centre for Structural Molecular Biology, School of Molecular & Cellular Biology, Faculty of Biological Sciences, University of Leeds, Leeds, United Kingdom; 2 Department of Microbiology and Immunology, University of Michigan Medical School, Ann Arbor, Michigan, USA; St. Jude Children's Research Hospital, Memphis, Tennessee, USA

**Keywords:** MNV, murine norovirus, CD300lf, receptor, membrane fluidity, virus evolution

## Abstract

Viruses interact with receptors on the cell surface to initiate and coordinate infection. The distribution of receptors on host cells can be a key determinant of viral tropism and host infection. Unravelling the complex nature of virus-receptor interactions is, therefore, of fundamental importance to understanding viral pathogenesis. Noroviruses are non-enveloped, icosahedral, positive-sense RNA viruses of global importance to human health, with no approved vaccine or antiviral agent available. Here, we use murine norovirus as a model to study the molecular mechanisms of virus-receptor interactions. We show that variation at a single amino acid residue in the major viral capsid protein, VP1 301, has a key impact on the interaction between virus and receptor. This variation did not affect virion replication or virus growth kinetics, but a specific amino acid was rapidly selected through evolution experiments and significantly improved cellular attachment when infecting cells in suspension. However, modulating plasma membrane mobility counteracted this phenotype, suggesting a role for membrane fluidity in norovirus cellular attachment. When the infectivity of a panel of recombinant viruses with single amino acid substitutions at this residue was compared *in vivo*, there were differences in the tissue distribution of viruses in a murine host, suggesting a role for VP1 301 in dissemination *in vivo*. Overall, these results highlight how capsid evolution can influence infectivity and dissemination in the host.

**IMPORTANCE:**

All viruses initiate infection by utilizing receptors to attach to target host cells. These virus-receptor interactions can therefore dictate viral replication and pathogenesis. Understanding the nature of virus-receptor interactions could also be important for the development of novel therapies. Noroviruses are non-enveloped icosahedral viruses of medical importance. They are a common cause of acute gastroenteritis with no approved vaccine or therapy and are a tractable model for studying fundamental virus biology. In this study, we utilized the murine norovirus model system to show that variation in a single amino acid of the major capsid protein alone can affect viral infectivity through improved attachment to suspension cells. Modulating plasma membrane mobility reduced infectivity, suggesting an importance of membrane mobility for receptor recruitment and/or receptor conformation. Furthermore, different substitutions at this site altered viral tissue distribution in a murine model, illustrating how in-host capsid evolution could influence viral infectivity and/or immune evasion.

## INTRODUCTION

The tissue distribution of virus infection is a key determinant of pathogenesis within a host and is dictated by several factors, including viral attachment to cellular receptors. Unravelling the complex nature of virus-receptor interactions can therefore make an important contribution to understanding viral pathogenesis. Human noroviruses (HNV) cause gastroenteritis and are responsible for >200,000 deaths and a cost of ~£40 billion worldwide each year (1). With no efficacious vaccine or approved therapy to treat HNV infections, a greater understanding of the virus life cycle and capsid structure is likely to be important for developing new approaches to disease control.

Noroviruses are members of the *Caliciviridae* family of positive-sense single-stranded RNA viruses (1) that have three or four open reading frames (ORF) 1–4 (2). ORF1 is translated to produce the viral polyprotein that is cleaved to generate the non-structural (NS) proteins required for genome replication (2). ORF2 and 3 encode the two viral structural proteins, VP1 and VP2, respectively (2). ORF4 is only expressed in murine norovirus (MNV) and encodes virulence factor 1 (VF1) (3). The two viral structural proteins assemble to enclose the genome in a *T = 3* capsid. This protein shell is ~40 nm in diameter and is composed of 180 copies (90 dimers) of the major structural protein VP1 and a low copy number of the minor structural protein VP2 (4). In feline calicivirus (FCV), 12 copies of VP2 form a portal-like assembly likely involved in genome release, but this is yet to be demonstrated for other caliciviruses (4). VP1 monomers comprise an N-terminal region, a shell domain, and a protruding domain. The protruding domain is additionally split into the proximal and distal sub-domains, P1 and P2, respectively (5, 6). *In vitro* replication of HNV has been demonstrated in human intestinal enteroids (7), human B cells (8), and salivary gland cells (9), but these models are technically challenging, are highly variable (10), and suffer from the lack of an effective reverse genetics system. Consequently, MNV is frequently used as a model system for the study of the norovirus structure and pathogenesis.

MNV is widely prevalent in laboratory mice (11). MNV-1 was the first strain of MNV to be identified (12), and it establishes acute, self-resolving infections in wild-type mice but can be fatal in immune compromised (STAT1^−/−^) mice (13). Different strains of MNV infect different site(s) within the host. Strains such as MNV-3 are located primarily in the colon and caecum (14), while MNV-1 is detected across the gastrointestinal tract and in immune cells (15), including macrophages and dendritic cells, which are thought to aid virus distribution to extra-intestinal sites (16–18). Furthermore, MNV-3 can be detected in the feces 56 days post-infection and can establish lifelong persistent infections (14). This draws parallels with HNV infection, whereby virus shedding can be detected up to 28 days post-infection (19), and persistent infection in immunocompromised individuals can last for years (20). Cellular tropism is also important in determining MNV persistence, with genotypes such as MNV-CR6 able to infect rare tuft cells located in the intestinal epithelium and evade the immune system (21, 22).

Cellular susceptibility to MNV is determined primarily by expression of CD300lf (the primary proteinaceous receptor) and CD300ld, while intracellular factors such as interferon-α/β receptor and STAT-1 also contribute to tropism through the restriction of viral replication (13, 23, 24). Both CD300lf and CD300ld are members of the CD300 receptor family of type I transmembrane proteins with a two disulfide-bonded extracellular domain (25). They are expressed on numerous immune cell types such as dendritic cells, where they are thought to play opposing roles in maintaining homeostasis (23, 26). Since the identification of CD300lf as the physiological receptor for MNV (27), the role of this interaction has been investigated. The P2 sub-domain of the VP1 capsid directly interacts with the receptor, with two CD300lf ectodomains binding one P2 sub-domain (24). The interaction mimics the way phospholipids bind to the receptor, is conserved across multiple MNV strains, and is enhanced by divalent cations (Ca^2+^ and Mg^2+^) and bile acid (24, 28, 29). Structural studies have suggested that up to 21 amino acids of VP1 form a network of interactions with 19 residues of CD300lf (5, 23, 24). Despite this extensive network of interactions, the binding affinity is reported to be low (*K*_D_: ~219 µM); therefore, receptor avidity may be important for endocytosis (24). Although some studies have investigated the role of VP1 genetic variation in pathogenesis (30–33), further research is required to fully understand the relative importance of each amino acid at the VP1 receptor interface.

Using the MNV model system, we demonstrate that variation in a single amino acid in the major capsid protein can alter virus-receptor interactions in cell culture, as well as dissemination in a mouse model. Specifically, our experiments suggest that a single substitution at this site can enhance infectivity in culture by increasing attachment to cellular receptors. Furthermore, modulation of cellular membrane mobility also reduced viral infection, suggesting a role of membrane fluidity in the recruitment of multiple receptors and/or for the correct CD300lf conformation. Finally, this amino acid variation affects MNV tissue distribution in mice, which has implications for viral dissemination within the host and organ-specific infection. Together, these results reveal how the plasticity of the viral capsid can affect cellular infectivity and could contribute to different infection phenotypes.

## RESULTS

### Identification of key residues in VP1 for MNV infectivity

**Fig 1:**
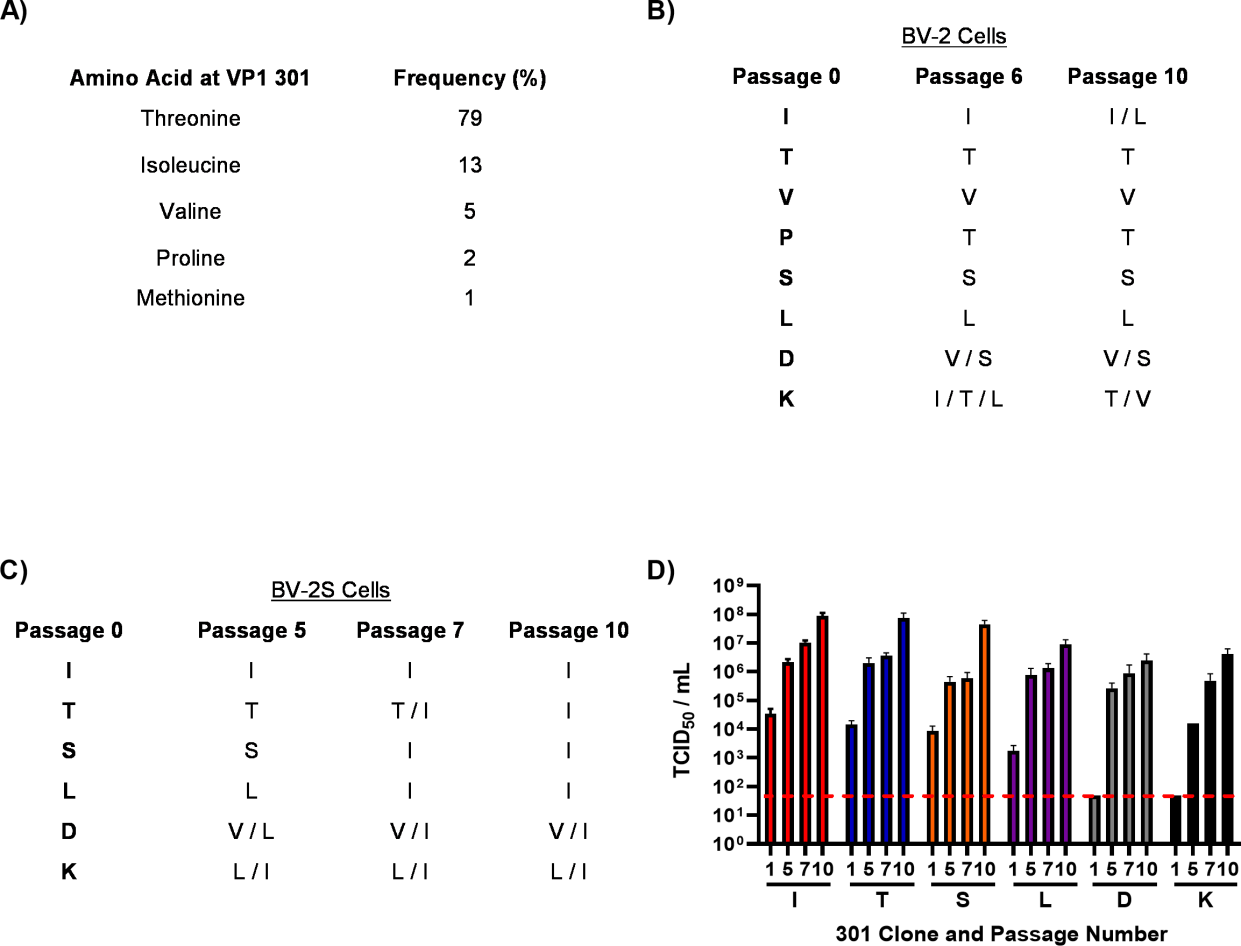
Sequential passaging of MNV-1 in suspension leads to selection of hydrophobic residues at VP1 301. (**A**) Overall amino acid variation at MNV VP1 301 was plotted from deposited sequences on GenBank. Recombinant MNV-1.CW1 with single amino acid substitutions in the infectious clones were passaged 10 times in (**B**) adherent BV-2 cells or (**C**) BV-2 cells in suspension (BV-2S), before ORF1, ORF2, ORF3, and ORF4 were sequenced at indicated passages. Data shows amino acid residues encoded at the 301 position of VP1 (*n* = 3). (**D**) Viruses passaged through BV-2S cells were titrated at selected passages as indicated. Red dotted line demonstrates limit of detection for TCID_50_ assay. Data show mean TCID_50_/mL (*n* = 3 ± SEM).

Previous studies identified 21 amino acids of MNV VP1 that form a network of interactions with the receptor CD300lf (23, 24, 34). Through alignment of all available MNV sequences, most of these residues are highly or completely conserved across MNV isolates; however, one residue, VP1 301, showed considerable variability (Fig. 1A). Furthermore, we noted an apparent correlation between the residue encoded in this position and viral strain assignment (Fig. S1A and B). For example, threonine (T) is predominant at VP1 301 in MNV-1, as well as MNV-4 and the persistent strains CR3 and CR6. Conversely, isoleucine (I) is predominant in MNV-5, MNV-6, and MNV-7 and the diarrheagenic WU23 strain. We therefore investigated how variations in the identity of this VP1 residue influence viral replication and pathogenesis.

We began by investigating whether variants at this amino acid position were genetically stable through cell culture evolution experiments. To ensure a homogenous genetic background, we modified an infectious clone of MNV-1.CW1 (that encodes T at amino acid 301 of VP1), to encode either I, valine (V), or proline (P). All of these amino acids have been documented at this position in MNV sequences deposited to GenBank. In order to ascertain the importance of the amino acid at this position, we also generated infectious clones coding for serine (S), leucine (L), aspartic acid (D), or lysine (K). S and L have similar biochemical properties to T and I, respectively, but different R group structure and so were chosen to investigate the relative importance of specific amino acids. Infectious clones were also modified to encode the charged amino acids D or K at VP1 301. These amino acids have not been identified at this position in natural isolates and therefore were predicted to be detrimental to viral fitness.

These clones were used to produce *in vitro*-transcribed RNA, and virus was recovered by transfection of BHK-21 cells (termed passage 0). The recovered viruses were serially passaged 10 times in BV-2 cells grown adherently or adapted for growth in suspension (for brevity termed BV-2S). Viruses were also passaged through the macrophage-like cell line RAW 264.7, as cells of this lineage are the most frequently used cell type for *in vitro* studies. RNA was extracted from virus samples taken at indicated passages, reverse transcribed, and the consensus genome sequence determined.

When passaged in adherent BV-2 cells (Fig. 1B), the VP1 sequence for the MNV-1.CW1 infectious clones carrying T301, V301, S301, and L301 did not change throughout the experiment. With the I301 infectious clone, two out of the three replicates maintained I301 at passage 10, while an I301L substitution occurred in the third replicate by passage 10. MNV-1.CW1 infectious clones carrying P301, D301, or K301 acquired substitutions at this position by passage 6 to encode a range of amino acids, which narrowed by passage 10 to P301T, D301V/S, and K301T/V. For all of the passaged viruses, no other amino acid changes were detected throughout ORF1, ORF2, ORF3, or ORF4. Furthermore, for MNV-1.CW1 T301 and MNV-1.CW1 I301, no amino acid substitutions in VP1 were identified when passaged through RAW 264.7 cells (Fig. S1C). In BV-2S cells, only the MNV-1.CW1 I301 clone was stable and did not acquire any VP1 amino acid substitutions throughout the experiment (Fig. 1C). In contrast, substitutions were found in MNV-1.CW1 T301, S301, and L301, which resulted in the selection of I at the consensus level (T301I, S301I, and L301I) between passages 5 and 7. Similarly, clones encoding D301 or K301 changed to D301V/I and K301L/I in the consensus sequence by passage 5. Importantly, there were no other changes to the ORF1, ORF2, ORF3, or ORF4 sequences.

To determine the effects of these substitutions on viral yield, the virus titers of supernatants from the BV-2S and RAW 264.7 cell passage experiments were assessed by TCID_50_ assay (Fig. 1D; Fig. S1D). The titer of the MNV-1.CW1 I301 clone increased over the duration of the BV-2S experiment from ~1 × 10^4^ TCID_50_/mL at passage 1 to ~1 × 10^8^ TCID_50_/mL by passage 10, which was the highest titer observed for any virus (Fig. 1D). Infectious clones carrying MNV-1.CW1 T301, S301, and L301 (that all changed to 301I) followed a similar pattern, having initial titers between 1 × 10^3^ and 1 × 10^4^ TCID_50_/mL, before increasing to ~1 × 10^7^ TCID_50_/mL by passage 10. The infectivity of MNV-1.CW1 D301 and K301 was below the limit of detection (LOD) until passage 5, when the titer increased to ~1 × 10^4^ and ~1 × 10^5^ TCID_50_/mL, respectively, before reaching a peak of ~1 × 10^7^ TCID_50_/mL at passage 10. This increase in titer coincided with the change to hydrophobic residues, with a preference for 301I. There was no difference in the titer of RAW 264.7-passaged MNV-1.CW1 I301 and T301 viruses throughout the duration of the experiment. Together, these data suggest that viruses with isoleucine at VP1 position 301 have a selective advantage when grown in suspension cell culture.

### The VP1 301 amino acid is a major determinant for infectious virus production in suspension cultures

**Fig 2:**
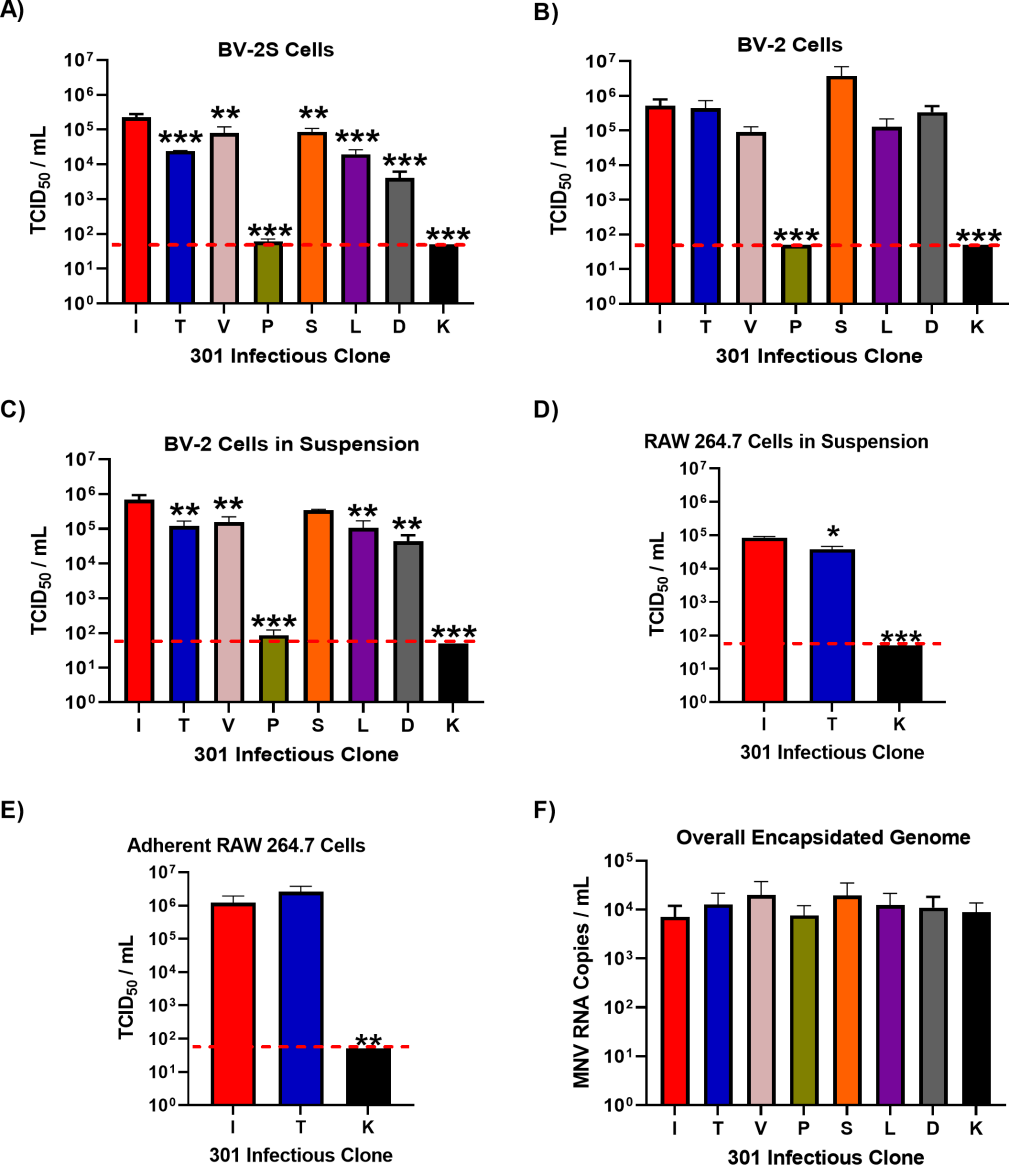
The VP1 301 residue is a major determinant of virus particle infectivity *in vitr*o. MNV-1.CW1 infectious clone RNAs with the indicated amino acids at VP1 301 were transfected into BHK-21 cells and virus-containing supernatants collected after 48 hours. Virus titer was determined by TCID_50_ assays on (**A**) suspension BV-2S cells, (**B**) adherent BV-2 cells, (**C**) adherent BV-2 cells infected in suspension, (**D**) RAW 264.7 cells infected in suspension, or (**E**) adherent RAW 264.7 cells. Data show mean TCID_50_/mL, with significance compared with I301 using one-way analysis of variance (ANOVA) with corrections for multiple comparisons (*n* = 3 ± SEM; *P < 0.05, **P < 0.01, and ***P < 0.001). The red dotted line denotes the limit of detection for TCID_50_ assays. (**F**) In separate transfections, the recovered supernatant was treated with 25 U/mL benzonase for 30 minutes at 37°C, before RNA was extracted and MNV genome copies were measured by one-step quantitative reverse transcription PCR (RT-qPCR). Data show mean MNV RNA genome copies/mL (*n* = 3 ± SEM).

To confirm that VP1 I301 conferred increased viral infectivity in suspension cell culture, the virus yield following transfection of BHK-21 cells with RNA was determined. RNA transcribed *in vitro* from the infectious clones was transfected into BHK-21 cells which are permissive for viral replication but do not express the viral receptor; therefore, the amount of infectious virus detected is directly proportional to the replication of the transfected RNA. Virus was collected and titrated by TCID_50_ assays on suspension-grown BV-2S cells (Fig. 2A), adherently grown BV-2 cells (Fig. 2B), or BV-2 cells grown adherently but infected in suspension (Fig. 2C). For suspension TCID_50_ assays, viral dilutions were added to the plates first before cells were seeded.

On BV-2S cells (Fig. 2A), the titer of the MNV-1.CW1 I301 variant was significantly higher than those for all other infectious clones. It was ~5-fold higher than MNV-1.CW1 V301 and S301 and ~10-fold greater than MNV-1.CW1 T301, L301, and D301. Both MNV-1.CW1 P301 and K301 variants had titers below LOD, showing that these substitutions are detrimental to MNV infectivity. In contrast, when the recovered viruses were titrated on adherently grown BV-2 cells (Fig. 2B), there were no significant differences in the titers of MNV-1.CW1 S301, I301, T301, V301, L301, and D301 viruses, with titers all between 1 × 10^5^ TCID_50_/mL and 1 × 10^6^ TCID_50_/mL. Again, MNV-1.CW1 P301 and K301 had greatly reduced infectivity.

To understand whether this observation was specific for cells grown or infected in suspension, the TCID_50_ assays were repeated with adherently grown BV-2 cells; however, the infection was performed while the cells were in suspension before being allowed to adhere to the culture vessels (Fig. 2C). In this setup, the titer of the MNV-1.CW1 I301 variant was again significantly higher than all other infectious clones, except MNV-1.CW1 S301, with both titers ~ 10-fold greater than for MNV-1.CW1 T301, V301, L301, and D301 variants. Once again, MNV-1.CW1 P301 and K301 viral recovery was at or below the LOD.

To rule out differences in transfection efficiency, we conducted similar experiments whereby infectious clone RNA was co-transfected into BHK-21 cells alongside an IRES-GFP DNA plasmid. The measurement of GFP fluorescence alongside virus yield allowed us to correct the viral titer for variation in transfection efficiency. The virus was titered by TCID_50_ assay in the same three cell infection conditions and normalized to GFP fluorescence at 24 hours post-transfection. Once again, MNV-1.CW1 I301 had a significantly greater titer compared with all other infectious clones when the TCID_50_ assay was conducted in BV-2S cells (Fig. S2A). There was no significant difference in viral titers between infectious clones in adherent BV-2 cells (Fig. S2B) and BV-2 cells infected in suspension (Fig. S2C).

To determine whether the observed differences between MNV-1.CW1 I301 and MNV-1.CW1 T301 viruses also applied to macrophages and B cells, cell types infected *in vivo* (13, 35), infectivity assays were performed in the macrophage-like RAW 264.7 cell line or suspension-grown mouse B lymphocyte cell line WEHI-231. RAW 264.7 cells were infected in suspension (Fig. 2D) or after adherence (Fig. 2E) and measured by TCID_50_ assay. While there was no difference between MNV-1.CW1 I301 and MNV-1.CW1 T301 recovery in the adherent infectivity assays, MNV-1.CW1 I301 had a significantly greater titer compared with MNV-1.CW1 T301 after infecting RAW 264.7 cells in suspension. MNV-1.CW1 K301 titer was below the LOD in both RAW 264.7 cell infectivity assays. For the WEHI-231 cell line, cells were infected as before and an MTS assay was used to determine residual cell viability and thus calculate virus infectivity (TCID_50_ assays could not be performed as WEHI-231 cells are semi-adherent; Fig. S2D). The titer of MNV-1.CW1 I301 was ~5-fold greater compared with MNV-1.CW1 T301. As a control for the WEHI-231 experiment, no virus was recovered from an infectious clone carrying a replication-defective mutation in the viral polymerase (GNN) (36).

To confirm that differences between cell types were not due to differences in replication rates, a one-step growth curve with MNV-1.CW1 T301 was carried out in BV-2, BV-2S, and RAW 264.7 cells. There was no statistically significant difference in viral titer throughout the experimental time course (Fig. S2E). To address potential differences in CD300lf expression between BV-2 and BV-2S cells, we compared the expression levels of the CD300lf receptor by flow cytometry (Fig. S2F). There was no difference in CD300lf receptor expression between BV-2 and BV-2S cells. It could be possible that the introduction of particular amino acids to the capsid at position VP1 301 (such as those not described in GenBank, i.e., K) may disrupt the replication or encapsidation of viral RNA, which could consequently account for differences in infectivity observed. To investigate this, the total production of viral particles was measured by quantifying RNase-protected viral genomes. Virus was produced from infectious clone RNA by transfection into BHK-21 cells as before, non-encapsidated RNA was then degraded by benzonase treatment before RNA was extracted and the protected RNA concentration was measured by one-step RT-qPCR (Fig. 2F). There were no statistically significant differences in the number of virus particles produced by any of the infectious clones, suggesting the variation in viral titer observed was not the result of reduced genome replication and encapsidation. Taken together, our data suggest that the MNV-1.CW1 I301 variant has a selective advantage at infecting cells when in suspension, but no selective advantage is observed in adherent cells.

### The amino acid at residue 301 in VP1 affects cell attachment *in vitro*

**Fig 3:**
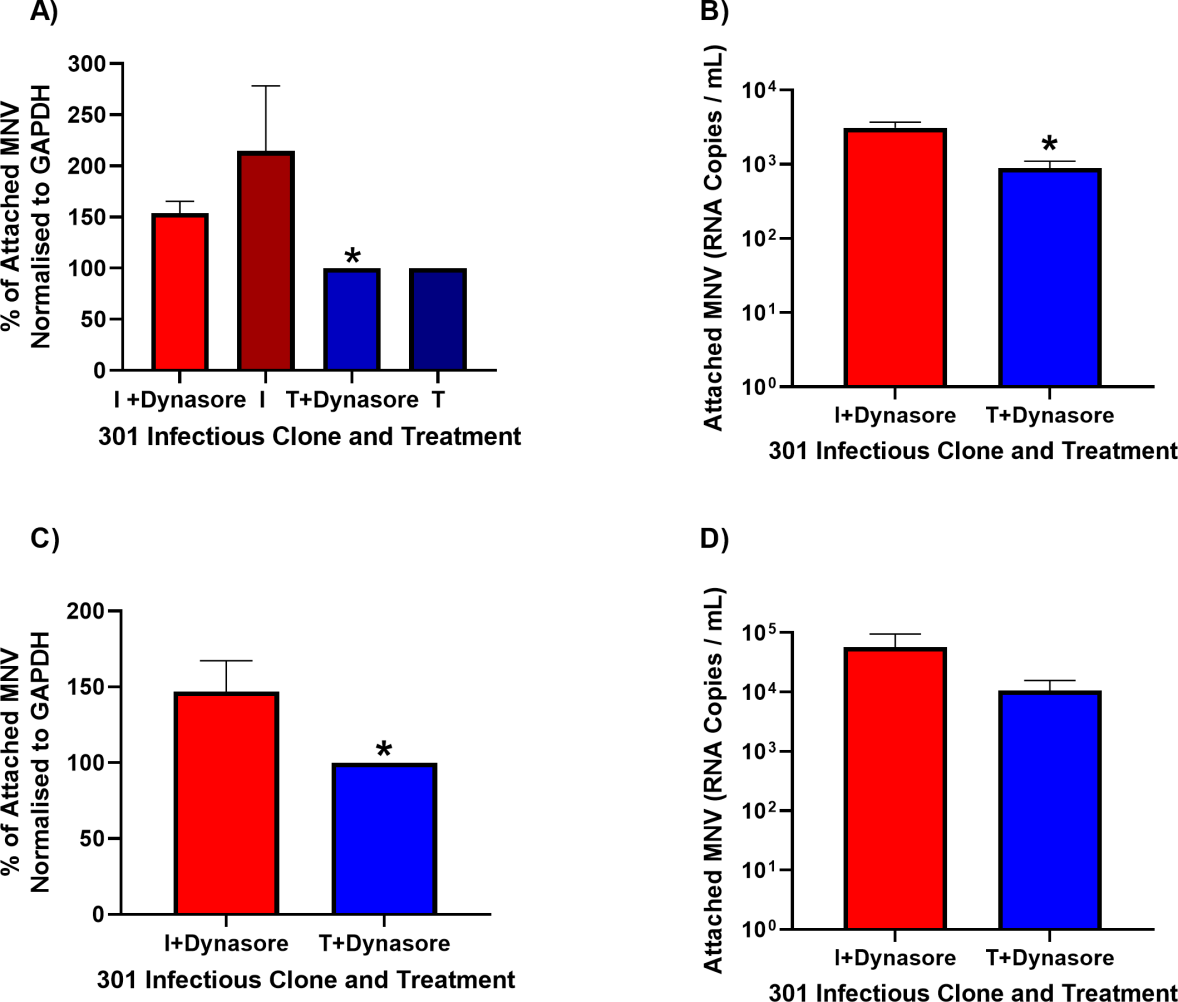
MNV-1.CW1 I301 demonstrates greater cell attachment *in vitro*. BV-2S or RAW 264.7 cells were untreated or pre-incubated with 50 µM dynasore (+Dynasore) for 30 minutes at 37°C, as indicated. Cells were subsequently incubated with MNV-1.CW1 I301 or MNV-1.CW1 T301 (multiplicity of infection [MOI] 10) for 2 hours at 37°C before the supernatant was removed and cells were pelleted and washed in ice-cold PBS. (**A**, **C**) Cell pellets were lysed with radioimmunoprecipitation assay (RIPA) buffer, and the amount of attached MNV was quantified by western blot for VP1 expression normalized to GAPDH expression for (**A**) BV-2S and (**C**) RAW 264.7 cells. Data show mean percentage increase/decrease for attached MNV-1.CW1 I301 compared with MNV-1.CW1 T301, with significance determined by unpaired *t*-test (*n* = 3 ± SEM, *P < 0.05). (**B**, **D**) Samples were treated with 25 U/mL benzonase for 30 minutes at 37°C, before RNA was extracted and MNV genome copies were measured by one-step RT-qPCR for (**B**) BV-2S and (**D**) RAW 264.7 cells. Data show mean genome copies per mL, with significance determined by unpaired *t*-test (*n* = 3 ± SEM, *P < 0.05).

VP1 residue 301 contributes to the virus-CD300lf receptor interface (24), and our data suggest that variation in this amino acid alone is sufficient to confer a replicative advantage to the virus. We hypothesized that the VP1 I301 variant has greater affinity for the receptor, thus increasing cell attachment. To investigate this hypothesis, we measured viral attachment with the MNV-1.CW1 I301 or T301 variants on BV-2S cells and RAW 264.7 cells in suspension (Fig. 3). We expected that the MNV-1.CW1 I301 variant would attach more effectively to cells in suspension compared with viruses encoding hydrophilic residues.

To prevent endocytosis, these assays were conducted on cells treated with dynasore, an inhibitor of dynamin that is required for MNV internalization (37). Cells were pre-treated with dynasore at 37°C or left untreated as controls, prior to incubation with MNV-1.CW1 I301 or T301 viruses. The amount of virus attached to the cells or remaining in the supernatant was measured by western blot for the major viral structural protein, VP1, normalized for GAPDH expression (in the cell-associated fraction) or BSA (for the supernatant faction, due to the presence of bovine serum in the media).

In both BV-2S and RAW 264.7 cells, significantly more MNV-1.CW1 I301 attachment was detected in the cell-associated fraction compared with MNV-1.CW1 T301 in the dynasore pre-treated cells (Fig. 3A and C; representative blot shown in Fig. S3A and B). To confirm these observations, we also quantified the amount of virus present in the cell-associated fraction by RT-qPCR for viral genomes. Briefly, non-encapsidated RNA was degraded by benzonase pre-treatment, before RNA was then extracted and MNV genome copy numbers in the cell-associated fraction were calculated by RT-qPCR. In concordance with the western blot results, in BV-2S there was significantly more MNV-1.CW1 I301 in the cell-associated fraction compared to MNV-1.CW1 T301 (Fig. 3B). The pattern of results was also the same for RAW 264.7 cells; however, the difference was not statistically significant (Fig. 3D).

### Membrane fluidity contributes towards MNV attachment *in vitro*

Taken together, our observations suggest that viruses encoding I301 have a selective growth advantage and increased cell attachment in suspension. As cells in suspension are reported to have a greater plasma membrane fluidity compared with adherent cells (38, 39), we hypothesized that fluidity of the cell membrane may affect viral attachment. We therefore sought to perturb membrane mobility first by measuring viral attachment at a reduced temperature (40, 41). As previously, BV-2S or RAW 264.7 cells were pre-treated with dynasore at 37°C, prior to incubation with MNV-1.CW1 I301 or T301 viruses at either 0°C or 37°C. The amount of virus attached to cells was measured as before by western blot and RT-qPCR.

**Fig 4:**
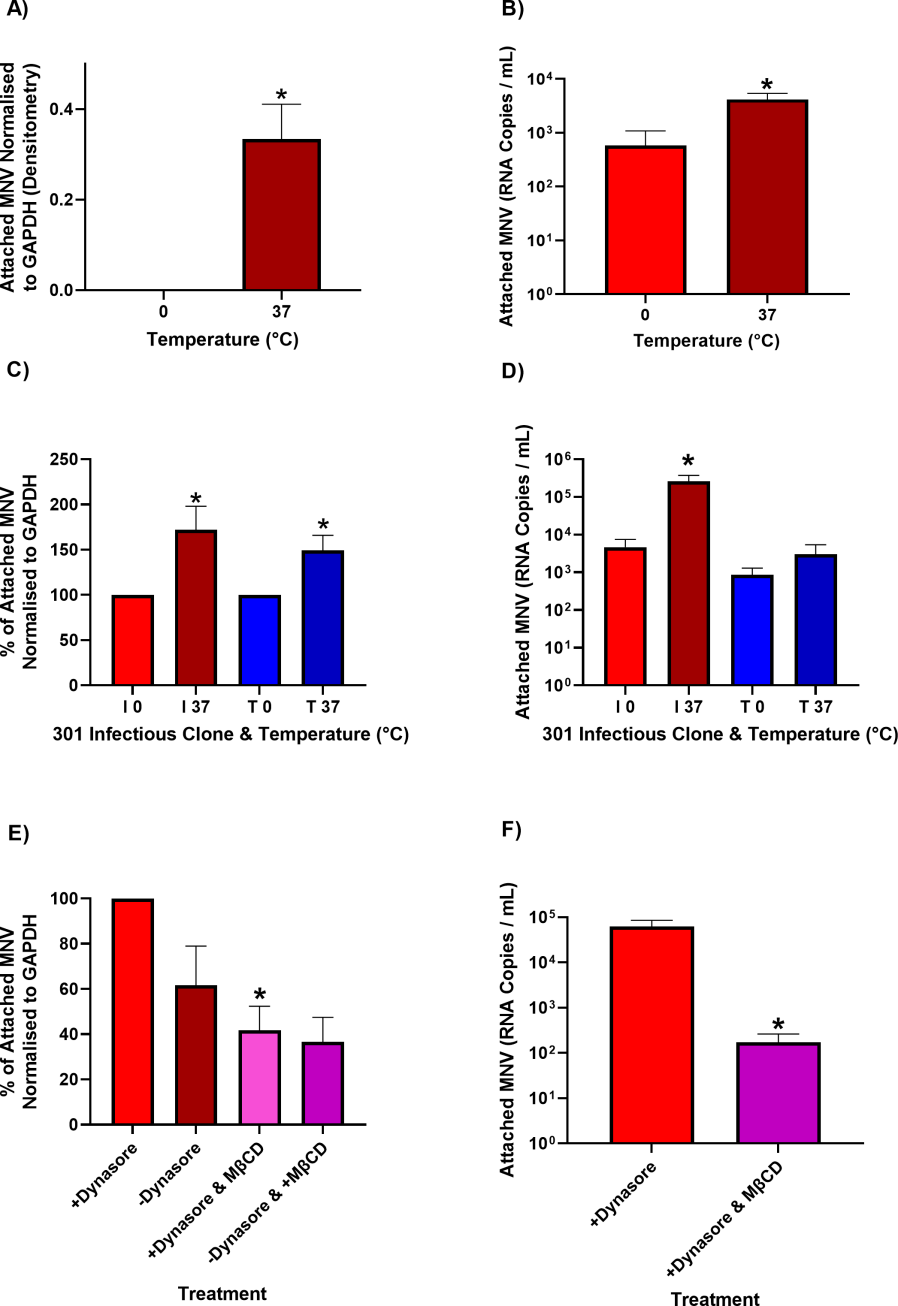
Membrane fluidity contributes to MNV attachment *in vitro*. BV-2S or RAW 264.7 cells were untreated or pre-incubated with 50 µM dynasore (+Dynasore) for 30 minutes at 37°C, as indicated. Cells were subsequently incubated with MNV-1.CW1 I301 or MNV-1.CW1 T301 (MOI 10) for 2 hours at 37°C or 0°C, as indicated before the supernatant was removed, and cells were pelleted and washed in ice-cold PBS. (**A**, **C**) Cell pellets were lysed with RIPA buffer, and the amount of attached MNV was quantified by western blot for VP1 expression normalized to GAPDH expression for (**A**) BV-2S and (**C**) RAW 264.7 cells. Data show mean percentage increase/decrease for attached MNV-1.CW1 I301 compared with MNV-1.CW1 T301, with significance determined by unpaired *t*-test (*n* = 3 ± SEM, *P < 0.05). (**B**, **D**) Samples were treated with 25 U/mL benzonase for 30 minutes at 37°C, before RNA was extracted and MNV genome copies were measured by one-step RT-qPCR for (**B**) BV-2S and (**D**) RAW 264.7 cells. Data show mean genome copies per mL, with significance determined by unpaired *t*-test (*n* = 3 ± SEM, *P < 0.05). (**E**, **F**) BV-2S cells were untreated or pre-incubated with 50 µM dynasore and/or 2 mM methyl-β-cyclodextrin (MβCD) for 60 minutes at 37°C, before incubation with MNV-1.CW1 I301 (MOI 10) for 2 hours at 37°C, the supernatant was removed, and cells were pelleted and washed in ice-cold PBS. (**E**) Cell pellets were lysed with RIPA buffer, and the amount of attached MNV was quantified by western blot for VP1 expression normalized to GAPDH expression. (**F**) Samples were treated with 25 U/mL benzonase for 30 minutes at 37°C, before RNA was extracted and MNV genomes copies were measured by one-step RT-qPCR. Data shows attached MNV normalized to GAPDH or mean genome copies per mL, with significance determined by unpaired *t*-test (*n* = 3 ± SEM, *P < 0.05).

In BV-2 cells at 0°C, there was significantly less attachment of MNV-1.CW1, such that little or no VP1 could be detected by western blot (Fig. 4A; representative blot shown in Fig. S3C). In agreement, by RT-qPCR, there was significantly less attachment of MNV-1.CW1 to BV-2S cells at reduced temperatures compared with 37°C. However, compared with the western blot data, MNV genomes were detected by RT-qPCR following attachment at 0°C in the cell-associated fraction, a difference likely due to the relative sensitivities of these methods (Fig. 4B). In RAW 264.7 cells, there was less attachment of both MNV-1.CW1 I301 and T301 at 0°C by western blot (Fig. 4C; representative blots shown in Fig. S3D and E) and RT-qPCR (Fig. 4D).

Next, these experiments were repeated after treating cells with MβCD, a compound reported to chemically perturb membrane mobility and lipid raft formation (42, 43). To conduct these experiments, BV-2S cells were untreated or pre-treated with MβCD with or without dynasore at 37°C prior to incubation with MNV-1.CW1 I301. As before, the amount of virus attached to the cells was quantified by western blot or RT-qPCR. By both western blot (Fig. 4E; representative blot shown in Fig. S3F) and RT-qPCR (Fig. 4F), MβCD significantly reduced MNV attachment in BV-2S cells. Finally, these experiments were repeated with losartan, a compound which can affect the behavior of membranes (44, 45). As previously, BV-2S cells were untreated or pre-treated with losartan with or without dynasore at 37°C prior to incubation with MNV-1.CW1 I301 and the amount of virus attached to the cells was quantified by western blot or RT-qPCR (Fig. S4A and B; representative blot shown in Fig. S3G). Treatment with losartan reduced MNV attachment by ~30% compared with untreated controls, consistent with membrane mobility as an important factor for MNV attachment.

To investigate the role of CD300lf conformation on MNV cell attachment in our assays, BV-2S cells were treated with myriocin. This is an inhibitor of serine palmitoyltransferase which is required for *de novo* sphingolipid biosynthesis, and sphingolipids have been demonstrated to be crucial in the MNV-CD300lf interaction by maintaining the correct receptor conformation (46).

To confirm the activity of myriocin, adherent BV-2 cells were untreated or treated with myriocin for 24 hours, before MNV-1.CW1 I301 was added for 2 hours. The supernatant was then removed, and cells were washed with PBS, before new media was added. Supernatant was then collected 12 hours later, and the titer of MNV was calculated by TCID_50_ assays on fresh BV-2 cells. There was a significant ~100-fold reduction in MNV titer in myriocin-treated cells (Fig. S4C). Next, the cell attachment assay was carried out as previously described, using MNV-1.CW1 I301. When analyzed by RT-qPCR, there was a significant reduction in attached MNV in the myriocin pre-treated cells (Fig. S4D).

Finally, to confirm that the various treatments did not affect cell viability, MTS assays were performed on BV-2S or RAW 264.7 cells after 2.5- or 24-hour treatment. As a positive control, cells were also treated with 90% dimethylsulfoxide (DMSO), which was anticipated to significantly reduce viability (Fig. S5). For both BV-2S and RAW 264.7 cells, cell viability was not significantly affected following incubation with dynasore, MβCD, losartan, and myriocin at the concentrations used for the relevant experimental timeframe. As expected, the 90% DMSO-positive control significantly reduced viability.

### The amino acid at residue 301 in VP1 influences MNV dissemination *in vivo*

**Fig 5:**
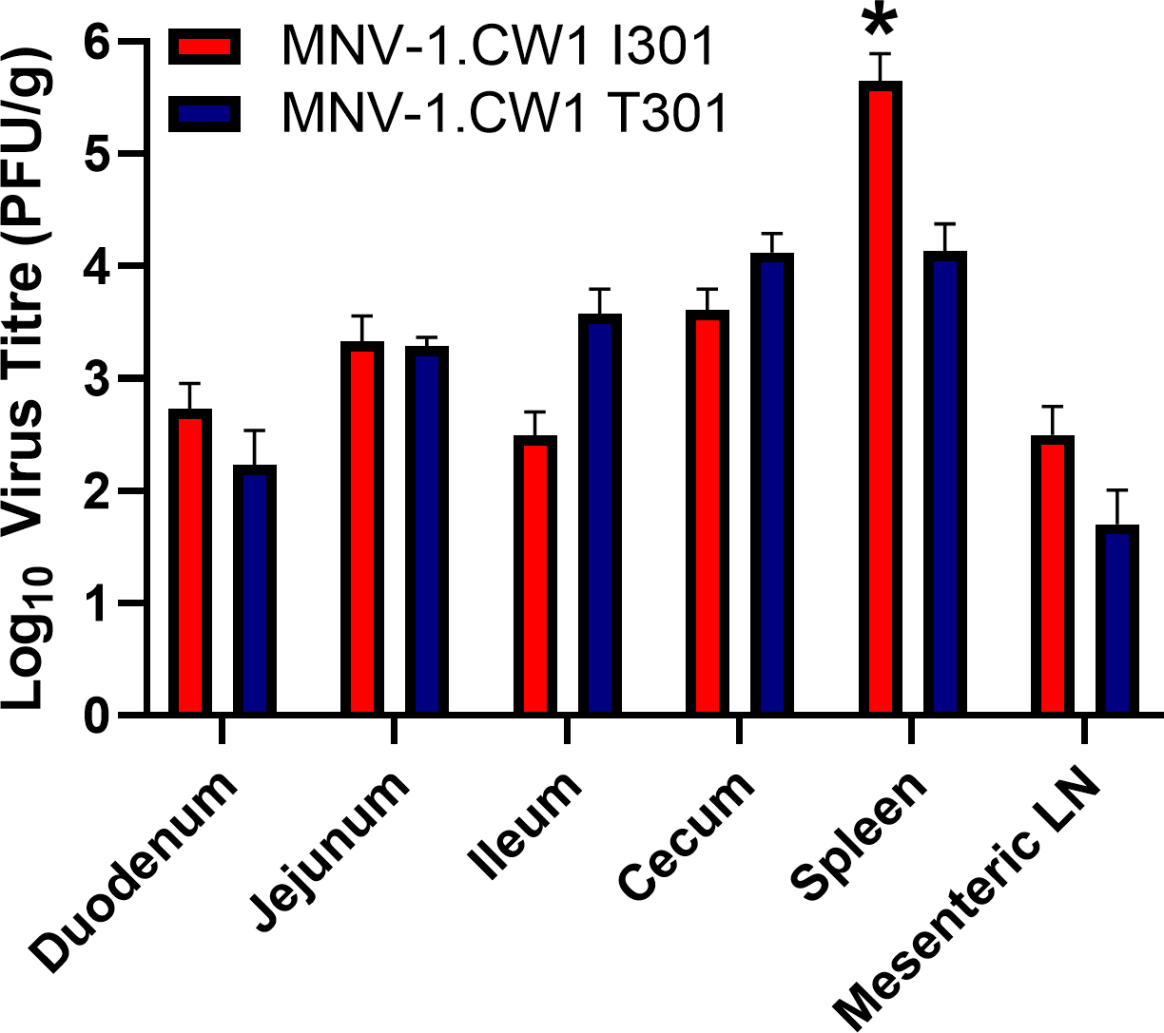
The VP1 residue 301 is a determinant of within-host spread in mice. Seven-week-old C57BL/6 mice were infected by oral gavage with 3 × 10^5^ PFU/mouse of MNV-1.CW1 I301 or MNV-1.CW1 T301. Mice were sacrificed at 12 hours post-infection, tissues were harvested, and viral titers were determined by plaque assay from the jejunum, duodenum, ileum, cecum, spleen, and mesenteric lymph nodes (LN). Plaque numbers (PFU) were normalized to tissue weight (in g). Data show mean PFU/g with significance compared with I301 using two-way ANOVA with corrections for multiple comparisons (*n* = 5 ± SEM, *P < 0.05).

Our data suggested that MNV-1.CW1 I301 infected suspension cells more effectively than viruses with other amino acids at this position. We therefore hypothesized that this variation at VP1 301 may affect virus dissemination in a murine model, due to improved infection of non-adherent immune cells at the early stages of infection. To investigate this, 7-week-old C57BL/6 mice were infected by oral gavage with 3 × 10^5^ PFU/mouse of MNV-1.CW1 I301 or T301. Tissues were harvested from the jejunum, duodenum, ileum, cecum, spleen, and mesentery lymph nodes at 12 hours post-infection, and MNV titers were measured by plaque assay (Fig. 5). The 12-hour time point represents the peak MNV-1 titer in C57BL/6 pups (47) and adult mice (48) and so was used to investigate the initial stages of infection, allowing host dissemination be assessed prior to immune clearance and before multiple rounds of MNV replication occurred.

Both MNV-1.CW1 I301 and T301 were detected in all organs sampled after 12 hours of infection. However, the MNV-1.CW1 I301 titer in the spleen was significantly higher compared with MNV-1.CW1 T301. To confirm the virus capsid had not undergone mutations during the mouse infection experiments, RNA was extracted from the spleen and the sequences of ORF2, ORF3, and ORF4 were determined. There were no changes to the ORF2, ORF3, or ORF4 consensus sequences compared with the input viruses. The mouse data agree with our cell culture experiments and suggest that the single amino acid substitution from T to I at position 301 in VP1 can lead to changes in viral dissemination in the native host.

## DISCUSSION

MNV has a tropism *in vivo* for adherent intestinal epithelial cells, as well as immune cells (13). MNV infection assays in cell culture are usually conducted on macrophage-like cells grown adherently, ignoring any consequences of infection of cells in suspension. Our work demonstrates that culture and passage of MNV-1 in suspension selects for a single amino acid substitution at VP1 301 that significantly increases infectivity, due to enhanced cell attachment *in vitro*. The experiments in mice reported here complement the cell culture data, with our findings demonstrating that MNV-1.CW1 I301 has increased dissemination to the spleen. Together, this work reveals the significance of residue 301 in VP1 in MNV-1 infectivity and dissemination.

Norovirus enters the gastrointestinal tract through transcytosis by M cells that are present at the epithelium of Peyer’s patches and at the tips of intestinal villi (49). Once inside the gastrointestinal tract, viruses come into contact with immune cells, such as dendritic cells and macrophages. The spleen is made up of red pulp and white pulp, which results in both a large reservoir of resident cells that mount adaptive response to pathogens, as well as being a site where damaged immune cells are phagocytosed (50). As such, many cell types are resident in the spleen, including dendritic cells (CD8a^+^, CD8a^−^, and monocyte-derived dendritic cells), monocytes, macrophages, and neutrophils (50). It has also been previously determined that following MNV-1 infection, there is an increase in macrophage and B-cell markers, with no change in dendritic cell marker expression (17). Our data suggest a mechanism whereby MNV-1.CW1 I301 has greater attachment and increased affinity to these cells, and thus, a greater proportion of this virus disseminates throughout the host via immune cells to the spleen and other organs. MNV-1.CW1 T301, on the other hand, attaches to and infects these cells less efficiently, and thus, more virus would stay proximal to the gastrointestinal tract. Limited sequence data deposited into GenBank correlate with this idea, with a number of studies demonstrating a consensus of T at position VP1 301 in virus sequenced from feces and persistent CR6 MNV that has a tropism for tuft cells in the intestinal tract (21, 32, 51, 52). Meanwhile, an I301 consensus was reported in VP1 in virus recovered from the brain and lymph nodes (12, 53). Our work is also consistent with findings in a previous study that demonstrated MNV-1.CW3 recruited monocytes and neutrophils, leading to increased immune cell numbers and viral loads in the spleen and mesenteric lymph nodes (18). While the capsid was found to be the determinant of this phenotype, the virus from the organs was not sequenced. Due to the plethora of CD300lf-rich cells present in the spleen, it is also possible that the VP1 variations not only influence dissemination but replication in this niche, which requires further investigation to elucidate.

The binding of MNV to CD300lf is thought to be a low-affinity, high-avidity interaction that requires a network of hydrophilic and hydrophobic interactions (24). In the X-ray crystal structure of VP1 protruding domains in complex with CD300lf, the residue 301 of VP1 is predicted to lie at the receptor interface (24). It is therefore possible that hydrophobic residues in this position increase the hydrophobic interactions required for receptor engagement and the initiation of infection. The results are consistent across BV-2S (CD300lf-lo), RAW 264.7, and WEHI-231 cell types (CD300lf-hi), which have differing levels of CD300lf expression (15, 23, 54). However, our data suggest that the relative preference for I301 is greater in BV-2S cells compared with RAW 264.7 cells, and therefore, the significance of this interaction may be greater in CD300lf-lo cells.

Each virion is thought to interact with multiple CD300lf receptors, forming clusters that increase avidity (24, 55). Our results are consistent with this hypothesis and suggest that membrane fluidity may play a role in this high avidity, with low temperature—which lowers membrane fluidity (40, 41)—resulting in decreased virus binding. Indeed, previous research has also suggested that macrophages can have increased plasma membrane mobility in response to pathogenic stimulus (56) or inflammation (57, 58).

These results build upon the established idea that cholesterol is required for MNV endocytosis and further implicates the importance of lipid rafts (37, 59), which contain both cholesterol and CD300lf (42, 43, 46, 60–62). As VP1 I301 enhances attachment to cells when in suspension, one interpretation of our data is that increased hydrophobic interactions between the VP1 I301 variant and CD300lf confer increased affinity with receptor clusters that form at cholesterol-rich areas of the membrane. A similar interaction occurs in other viruses, such as influenza, where cholesterol induces nano-clusters of the glycosphingolipid receptor to increase virus infectivity (63). Previous work has also shown that GCDCA and metal ions can induce MNV protruding domain conformational changes that increase receptor affinity (6, 28), while other research has suggested the importance of sphingolipid biosynthesis in CD300lf receptor conformation to facilitate MNV infection (46). Consistent with this study, sphingolipid depletion in BV-2S reduced MNV attachment. Future work should therefore determine the relative roles of membrane mobility, lipid rafts, and receptor/ VP1 conformation *in vitro* and *in vivo*.

The results described here suggest that I301 plays a physiologically important role. It must be noted, however, that a previous study suggested the T301I substitution is a tissue culture adaptation, with MNV-3 collected from mice feces 56 days post-infection reverting to T301 (15), which we did not observe in our experiments. This would be consistent with our hypothesis that viruses that shed in the feces predominantly encode T301, while those that spread to the lymphoid organs predominantly have I301 at this position. The difference in these findings, therefore, may be explained by experimental variations such as the sequenced tissue site and/or time post-infection. Furthermore, a recent study demonstrated the diarrheagenic potential of MNV-WU23 in neonatal pups (47). MNV-WU23 can infect cells in the intestine, in addition to cells in the extra-intestinal tissues (including spleen), and was demonstrated to have six P2 substitutions, compared with MNV-CR6, including VP1 T301I (47). In this study, it was determined that VP1 I301 alone was not sufficient to cause diarrheagenic pathogenesis, and our data complement this report, indicating it may instead play a role in the extra-intestinal spread.

During infection of the host, viral quasi-species may provide the VP1 sequence diversity to generate viral sub-populations that allow access to different host cell types and widen dissemination. Furthermore, these sub-populations may change over time, depending on host immune pressures. Indeed, the T301I substitution has been previously identified as one of three mutations that occurred in an MNV escape mutant, following the addition of a monoclonal antibody that targeted VP1 (64). Viral quasi-species evolution is likely to be of relevance to HNV pathogenesis and chronic infection. Chronic HNV infection can persist for years in immunocompromised patients, leading to dehydration and nutrient deficiencies that can lead to mortality (19, 20, 65). Evolutionary studies have shown that HNV amino acid mutations accumulate throughout the chronic infection period (66, 67), with most being located in the VP1 protruding domain (67). These evolutionary changes have also been linked to immune evasion, which leads to changes in antigenic epitopes of the virus (68). However, data that utilize virus-like particles and bioinformatics are contradictory as to whether this can change receptor-binding interactions (68, 69). Our study demonstrates a mechanism by which virus capsid evolution can alter the phenotype of infection by enhancing interaction with the host cell. It can be postulated that this mechanism may also be utilized by HNV to avoid immune detection and influence chronic infection. This hypothesis should be investigated further as current reverse genetics systems (70, 71) are improved to allow the study of HNV infectivity.

## MATERIALS AND METHODS

### Cells and mice

BHK-21 cells (obtained from ATCC) and RAW 264.7 cells (gifted by Ian Clarke, University of Southampton) were maintained as previously described (6) with adherently grown BV-2 cells (gifted by Ian Goodfellow, University of Cambridge), maintained using the same method. Suspension-grown BV-2 cells (referred to as BV-2S cells) were cultured in spinner flasks by maintaining a viable density of 0.5–1 × 10^6^ cells/mL with medium changes every 2 days. WEHI-231 cells (obtained from ATCC) were maintained as previously described (72). Cells were incubated at 37°C and 5% CO_2_.

Balb/c mice were purchased from Jackson Laboratories (Bar Harbor, ME) and housed under specific pathogen-free (SPF) and MNV-free conditions in accordance with federal and university guidelines. The protocol was approved by the University of Michigan Committee on Use and Care of Animals (UCUCA protocol number PRO00010484). Mice were allowed to acclimate in the facility for 6 days prior to infection. Mice were infected via oral gavage with 3 × 10^5^ PFU of virus in 200 µL/mouse. Tissues were harvested at 12 hours post-infection and processed for plaque assay as described (73).

### Plasmid constructs

The plasmid, pT7-MNV*, containing the infectious clone sequence from MNV-1 strain CW1P3 (37) under control of T7 promoter was used for virus recovery (74). To exchange VP1 T301, standard two-step overlapping PCR mutagenesis was used with this plasmid as a template (31). The pcDNA3.1(+)IRES GFP plasmid used for transfection experiments (kindly donated by Jamel Mankouri, University of Leeds) has already been described (75). Sequences of plasmids and primers are available on request.

### *In vitro* transcription and virus recovery

MNV plasmids were linearized with *Not*I and phenol/chloroform extracted before being used for *in vitro* transcription using the HiScribe T7 ARCA mRNA Kit (NEB), following the manufacturer’s instructions. RNA was purified and concentrated using the RNA Clean and Concentrator Kit (Zymo). RNA/DNA transfection was carried out as previously described (76). GFP fluorescence at 24 and 48 hours post-transfection was analyzed via the Incucyte S3 machine (Sartorius).

### TCID_50_ assay

Infectious viral titer was determined using a TCID_50_ assay modified from Hwang et al. (77), as per reference (6). For adherent TCID_50_ assays, BV-2 cells were seeded into 96-well plates at 2 × 10^4^ cells/well and left overnight before infection. For suspension TCID_50_ assays, viral dilutions were prepared and added to the plates first, prior to infection. TCID_50_ values were calculated according to the Spearman and Kärber algorithm (78).

### MTS assay

Cell viability in WEHI-231 cells was calculated 48 hours after MNV infection via the CellTiter 96 AQueous One Solution Cell Proliferation Assay Kit, following manufacturer’s instructions. Absorbance was read on the Infinite F50 (Tecan) machine. Cytopathic effect was assigned to wells with values under 1. The number of positive wells was then used to calculate TCID_50_ values. Cell viability in BV-2 cells and RAW 264.7 cells was calculated 2.5 hours or 24 hours after treatment with 50 µM dynasore, 2 mM MβCD, 10% PBS, 10% DMSO, 10% methanol, or 90% DMSO via the CellTiter 96 AQueous One Solution Cell Proliferation Assay Kit, following the manufacturer’s instructions. Absorbance was read on the Infinite F50 (Tecan) machine.

### Plaque assay

The plaque assay was performed from virus isolated from mouse tissue as previously described (73, 79). Data were normalized to the tissue weight and expressed as PFU per gram of tissue.

### MNV RNA extraction and sequencing

Viral RNA was extracted using the Direct-zol RNA Miniprep Kit (Zymo Research) according to the manufacturer’s instructions. For genome sequencing, ORF1, ORF2, ORF3, and ORF4 were amplified by RT-PCR using Superscript IV (Invitrogen), followed by second-strand synthesis using Phusion DNA Polymerase (NEB). The sequence of the amplicon was determined by Sanger sequencing (Azenta). Sequences of primers used are available on request.

### One-step RT-qPCR

Virus sample was treated with 25 U/mL recombinant HS-Nuclease (MoBiTec) at 37°C for 30 minutes, and viral RNA was extracted as previously described (76). RNA reverse transcription and cDNA amplification were then carried out by the GoTaq 1-Step RT-qPCR System (Promega), using established primers (80). CT values were converted to RNA copies/mL by analyzing against a pT7-MNV* RNA standard curve of known values. The results were read using the Stratagene Mx3005P qPCR machine (Agilent Technologies).

### Western blot

SDS-PAGE and western blot analysis were carried out as previously described (81). Primary antibodies used were anti-MNV VP1 monoclonal antibody (MABF2097, Sigma-Aldrich), anti-CD300lf monoclonal antibody (MAB27881, R&D Systems), anti-GAPDH monocolonal antibody (60004-1, ProteinTech), and anti-BSA monoclonal antibody (66201-1, ProteinTech). Polyclonal anti-mouse (PA1-84388, Invitrogen) and anti-rabbit (HAF008, R&D Systems) HRP conjugates were employed as a secondary antibody. Blots were analyzed on the G:BOX Chemi XX6 machine (Syngene) and densitometry calculated using ImageJ.

### Flow cytometry

Detached adherent BV-2 cells or BV-2S cells (2 × 10^6^/mL) were analyzed for CD300lf expression using a flow cytometry protocol previously described (27), with anti-CD300lf primary antibody (MAB27881, R&D Systems) and Alexafluor647 goat Anti-mouse IgG (A-21235, Invitrogen). The samples were analyzed on a Cytoflex S flow cytometer (Beckman Coulter).

### Viral attachment assay

Viral attachment to BV-2S or RAW 264.7 cells was determined using an attachment assay modified from Berry and Tse (82). 10^5^ BV-2S cells were pre-treated with 50 µM dynasore (Cambridge Bioscience), 2 mM MβCD (Sigma-Aldrich), and/or 40 mM losartan (Sigma-Aldrich) for 30 minutes at 37°C. MNV was added to the cells at an MOI of 10 or 1 (as indicated) and incubated at either 0°C or 37°C for 2 hours, before completing the assay as described. For myriocin assays, cells were pre-treated with 25 µM myriocin (Cambridge Bioscience) for 24 hours, prior to dynasore treatment. To confirm the activity of myriocin, adherent BV-2 cells were pre-treated with 25 µM myriocin for 24 hours, before virus was added for 2 hours. Supernatant was then replaced after a PBS wash, before cells were incubated for a further 12 hours. The supernatant was then collected and MNV titer calculated by TCID_50_ assay on fresh BV-2 cells.

### Statistics

Data were analyzed and presented using GraphPad Prism v9.0 as mean ± SEM, *N*—biological repeat, with the number of repeats stipulated in the figure legends. Statistical tests performed are also detailed within the figure legends with significant differences indicated by *P < 0.05, **P < 0.01, and ***P < 0.001.

## SUPPLEMENTAL MATERIAL

Figure S1 jvi.01719-23-s0001.docx Association between VP1 301 and MNV strain specificity.

Figure S2 jvi.01719-23-s0002.docx The VP1 I301 residue is a determinant of virus infectivity *in vitro*.

Figure S3 jvi.01719-23-s0003.docx Representative Western blots from cell attachment assays with results shown in Figures 3 and 4.

Figure S4 jvi.01719-23-s0004.docx Losartan and myriocin reduces MNV attachment in BV-2 cells.

Figure S5 jvi.01719-23-s0005.docx Dynasore, MβCD, losartan and myriocin did not significantly reduce cell viability.

ASM does not own the copyrights to Supplemental Material that may be linked to, or accessed through, an article. The authors have granted ASM a non-exclusive, world-wide license to publish the Supplemental Material files. Please contact the corresponding author directly for reuse.
