# Supplementary material for: Amino acid substitutions in norovirus VP1 dictate host dissemination via variations in cellular attachment
Source: J Virol. 2023 Nov 30;97(12):e01719-23. doi: 10.1128/jvi.01719-23 (PMC10734460; doi:10.1128/jvi.01719-23)
Supplement: Figure S1 — Association between VP1 301 and MNV strain specificity. [file jvi.01719-23-s0001.docx]

**Supplemental Figure 1: Association between VP1 301 and MNV strain specificity. (A)** The frequency of consensus isoleucine or threonine present at VP1 301 in different MNV strains was plotted from deposited sequences on GenBank. MNV-1, n = 13; MNV-3, n = 4; MNV-2, MNV-4, CR6, WU20, n = 2; MNV-5, MNV-6, MNV-7, CR3, S9, WU23, WU25, WU26, n = 1. **(B)** The consensus amino acid at MNV VP1 301 from deposited sequences on GenBank was plotted corresponding to the location the virus was isolated and sequenced from (when specified). Faeces, n = 6; brain and lymph, n = 1. **(C)** Recombinant MNV-1.CW1 with either threonine or isoleucine at position VP1 301 were passaged 10 times in RAW 264.7 cells, before ORF2 was sequenced at indicated passages. Data shows amino acid residues encoded at the VP1 301 position (n = 2). **(D)** MNV-1.CW1 I301 and MNV-1.CW1 T301 passaged through RAW 264.7 cells were titrated. Red dotted line demonstrates limit of detection for TCID_50_ assay. Data shows mean TCID_50_/mL (n = 2 ± SEM).
