# Supplementary material for: Amino acid substitutions in norovirus VP1 dictate host dissemination via variations in cellular attachment
Source: J Virol. 2023 Nov 30;97(12):e01719-23. doi: 10.1128/jvi.01719-23 (PMC10734460; doi:10.1128/jvi.01719-23)
Supplement: Figure S2 — The VP1 I301 residue is a determinant of virus infectivity in vitro. [file jvi.01719-23-s0002.docx]

**Supplemental Figure 2: The VP1 I301 residue is a determinant of virus infectivity *in vitro*.** MNV-1.CW1 infectious clone RNAs with the indicated amino acids at VP1 301 were transfected into BHK-21 cells alongside an IRES-GFP plasmid and virus-containing supernatants collected after 48 hours. Virus titre was determined by TCID_50_ assays on **(A)** BV-2S cells, **(B)** adherent BV-2 cells or **(C)** adherent BV-2 cells infected in suspension and normalised to GFP fluorescence at 24 hours. Data shown as change in infectivity compared to I301, with significance compared to I301 using one-way ANOVA with corrections for multiple comparisons (n = 3 ± SEM; **p<0.01, **p<0.001). **(D)** MNV-1.CW1 infectious clone RNAs with the indicated amino acids at VP1 301 were transfected into BHK-21 cells and virus-containing supernatants collected after 48 hours. Virus titre was determined by MTS assay on WEHI-231 B lymphocyte suspension cells. The experiment also contained an RdRp replication-defective MNV (GNN) negative control. Data shows mean TCID_50_/mL, with significance compared to I301 using one-way ANOVA with corrections for multiple comparisons (n = 3 ± SEM; **p<0.01; ***p<0.001). **(E)** One-step growth curves of MNV-1.CW1 (T301) in BV-2, BV-2S and RAW 264.7 cells, infected at 10 PFU/cell. No significant differences were observed in titres between the cell types at each time point using the one-way ANOVA with corrections for multiple comparisons; data ± SEM, n = 3. **(F)** CD300lf expression in the indicated cells was measured by flow cytometry (with negative no primary antibody controls; n = 2; representative plot shown).
