# Supplementary material for: Amino acid substitutions in norovirus VP1 dictate host dissemination via variations in cellular attachment
Source: J Virol. 2023 Nov 30;97(12):e01719-23. doi: 10.1128/jvi.01719-23 (PMC10734460; doi:10.1128/jvi.01719-23)
Supplement: Figure S3 — Representative Western blots from cell attachment assays with results shown in Figures 3 and 4. [file jvi.01719-23-s0003.docx]

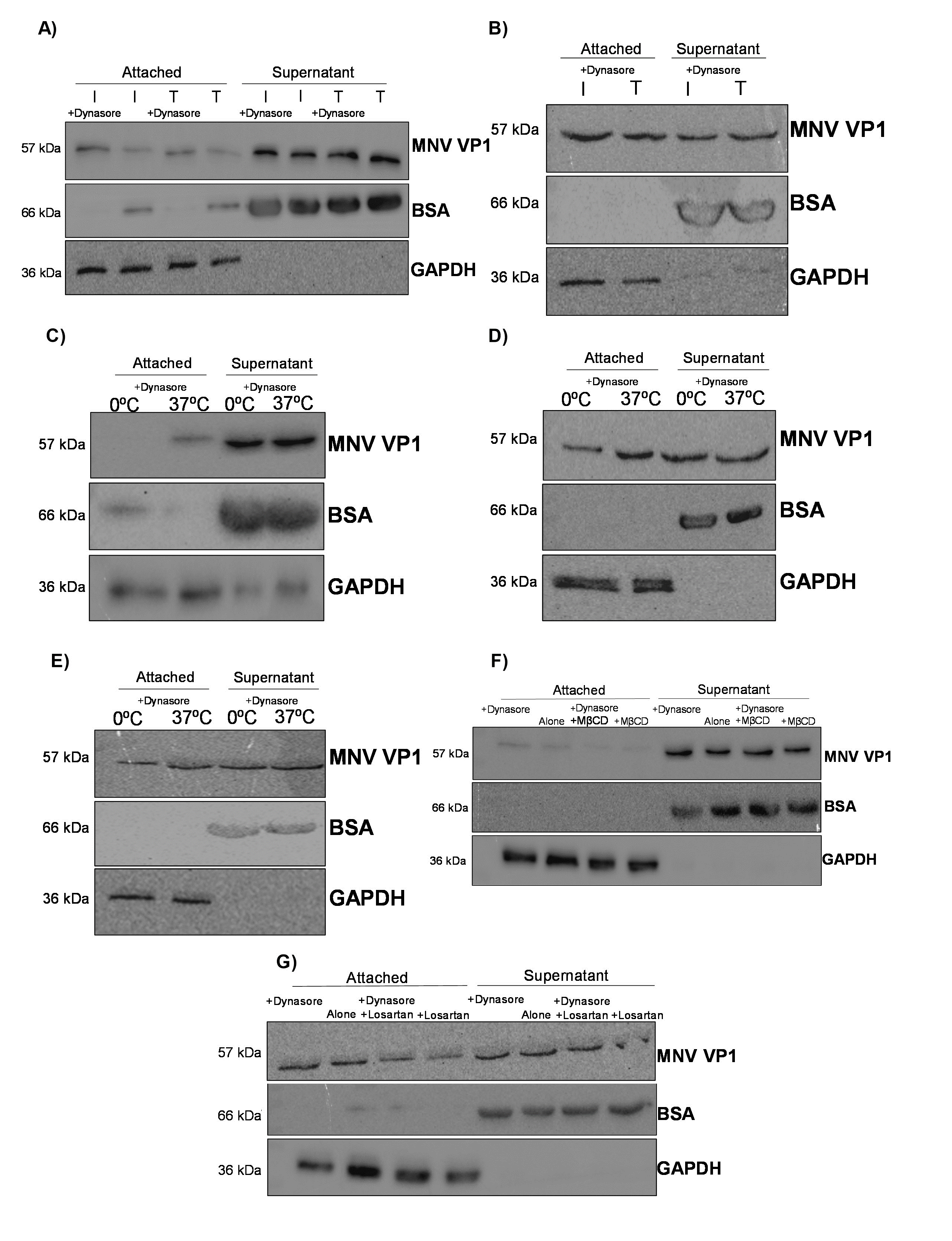
**Supplemental Figure 3: Representative western blots from cell attachment assays performed in Figures 3 and 4. (A and C)** BV-2S or **(B, C and E)** RAW 264.7 cells were untreated or pre-incubated with 50 µM dynasore (+Dynasore) for 30 minutes at 37⁰C, as indicated. Cells were subsequently incubated with MNV-1.CW1 I301 or MNV-1.CW1 T301 (MOI 10) for 2 hours at 0⁰C or 37⁰C as indicated, before the supernatant was removed, cells pelleted and washed in ice cold PBS. **(F and G)** BV-2S cells were untreated or pre-incubated with 50 µM dynasore and/or 2 mM **(F)** methyl-β-cyclodextrin (MβCD) or **(G)** 40 mM losartan for up to 60 minutes at 37⁰C, MNV-1.CW1 I301 (MOI 10) was then added for 2 hours at 37⁰C, before the supernatant was removed, cells pelleted and washed in ice cold PBS. All cell pellets were lysed with RIPA buffer and the amount of MNV present in each fraction was quantified by western blot with GAPDH and BSA used as loading controls. One representative blot for all experiments shown.
