# Supplementary material for: Amino acid substitutions in norovirus VP1 dictate host dissemination via variations in cellular attachment
Source: J Virol. 2023 Nov 30;97(12):e01719-23. doi: 10.1128/jvi.01719-23 (PMC10734460; doi:10.1128/jvi.01719-23)
Supplement: Figure S4 — Losartan and myriocin reduces MNV attachment in BV-2 cells. [file jvi.01719-23-s0004.docx]

**Supplemental Figure 4: Losartan and Myriocin reduces MNV attachment in BV-2 cells.** BV-2S cells were untreated or pre-incubated with 50 µM dynasore and/or 40mM Losartan for 60 minutes at 37⁰C, before incubation with MNV-1.CW1 I301 (MOI 10) for 2 hours at 37^o^C. The supernatants were then removed, cells pelleted and washed in ice cold PBS. **(A)** Cell pellets were lysed with RIPA buffer and the amount of attached MNV was quantified by western blot for VP1 expression normalised to GAPDH expression. **(B)** Samples were treated with 25 U/mL benzonase for 30 minutes at 37⁰C, before RNA was extracted and MNV genomes copies measured by one-step RT-qPCR. **(C)** BV-2 cells were untreated or pre-incubated with 25 µM myriocin for 24 hours at 37⁰C before MNV-1.CW1 I301 at an MOI of 1 was added for 2 hours. The supernatants were then removed and cells washed with PBS before new media was added and the cells further incubated at 37⁰C for 16 hours. Supernatants were collected and the titres of MNV were calculated by TCID_50_ assays on fresh BV-2 cells. **(D)** BV-2 cells were untreated or pre-incubated with 25 µM myriocin for 24 hours at 37⁰C, before cells were detached by trypsin, washed in PBS and then pre-incubated with 50 µM dynasore for 30 minutes at 37⁰C. Cells were subsequently incubated with MNV-1.CW1 I301 (MOI 1) for 2 hours at 37⁰C, before the supernatants were then removed, cells pelleted and washed in ice cold PBS. Samples were treated with 25 U/mL benzonase for 30 minutes at 37⁰C, before RNA was extracted and MNV genome copies measured by one-step RT-qPCR. Data shows mean MNV titre or RNA copies per mL for attached MNV, with significant differences in untreated and treated cells demonstrated using unpaired T-test (n = 3 ± SEM, *p<0.05; ***p<0.001).
