# Supplementary material for: Amino acid substitutions in norovirus VP1 dictate host dissemination via variations in cellular attachment
Source: J Virol. 2023 Nov 30;97(12):e01719-23. doi: 10.1128/jvi.01719-23 (PMC10734460; doi:10.1128/jvi.01719-23)
Supplement: Figure S5 — Dynasore, MβCD, losartan and myriocin did not significantly reduce cell viability. [file jvi.01719-23-s0005.docx]

**Supplemental Figure 5: Dynasore, MβCD, losartan and myriocin did not significantly reduce cell viability. (A)** BV-2 cells or **(C)** RAW 264.7 cells were untreated or incubated with either 50 µM dynasore, 2 mM methyl-β-cyclodextrin (MβCD), 40 mM losartan, 10% PBS, 10% dimethylsulfoxide (DMSO) or 90% DMSO in the indicated combinations for 2.5 hours, before cell viability was measured by MTS assay. **(B)** BV-2 cells or **(D)** RAW 264.7 cells were untreated or incubated with either 25 µM myriocin, 10% methanol, or 90% DMSO for 24 hours, before cell viability was measured by MTS assay. Data show mean percentage cell viability normalised to untreated controls, with significance compared to untreated using one-way ANOVA with corrections for multiple comparisons (n = 3 ± SEM, **p<0.01,***p<0.001).
